# Structural Olfactory Nerve Changes in Patients Suffering from Idiopathic Intracranial Hypertension

**DOI:** 10.1371/journal.pone.0035221

**Published:** 2012-04-06

**Authors:** Christoph Schmidt, Edzard Wiener, Jan Hoffmann, Randolf Klingebiel, Felix Schmidt, Tobias Hofmann, Lutz Harms, Hagen Kunte

**Affiliations:** 1 Institute of Radiology, Charité-Universitätsmedizin Berlin, Berlin, Germany; 2 Department of Neurology, Charité-Universitätsmedizin Berlin, Berlin, Germany; 3 Department of Psychosomatic Medicine, Charité-Universitätsmedizin Berlin, Berlin, Germany; Banner Alzheimer’s Institute, United States of America

## Abstract

**Background:**

Complications of idiopathic intracranial hypertension (IIH) are usually caused by elevated intracranial pressure (ICP). In a similar way as in the optic nerve, elevated ICP could also compromise the olfactory nerve system. On the other side, there is growing evidence that an extensive lymphatic network system around the olfactory nerves could be disturbed in cerebrospinal fluid disorders like IIH. The hypothesis that patients with IIH suffer from hyposmia has been suggested in the past. However, this has not been proven in clinical studies yet. This pilot study investigates whether structural changes of the olfactory nerve system can be detected in patients with IIH.

**Methodology/Principal Findings:**

Twenty-three patients with IIH and 23 matched controls were included. Olfactory bulb volume (OBV) and sulcus olfactorius (OS) depth were calculated by magnetic resonance techniques. While mean values of total OBV (128.7±38.4 vs. 130.0±32.6 mm^3^, *p*=0.90) and mean OS depth (8.5±1.2 vs. 8.6±1.1 mm, *p*=0.91) were similar in both groups, Pearson correlation showed that patients with a shorter medical history IIH revealed a smaller OBV (r=0.53, *p*<0.01). In untreated symptomatic patients (n=7), the effect was greater (r=0.76, *p*<0.05). Patients who suffered from IIH for less than one year (n=8), total OBV was significantly smaller than in matched controls (116.6±24.3 vs. 149.3±22.2 mm^3^, *p*=0.01). IIH patients with visual disturbances (n=21) revealed a lower OS depth than patients without (8.3±0.9 vs. 10.8±1.0 mm, *p*<0.01).

**Conclusions/Significance:**

The results suggest that morphological changes of the olfactory nerve system could be present in IIH patients at an early stage of disease.

## Introduction

Idiopathic intracranial hypertension (IIH) is characterized by increased intracranial pressure (ICP) and is affecting mainly obese women of childbearing age. The aetiology of the disorder is not well understood but disturbed cerebrospinal fluid (CSF) dynamics are assumed to be an important factor. Affected patients mostly suffer from chronic disabling headache and other symptoms of elevated ICP like visual disturbance, tinnitus and diplopia. Impairment of visual function is often progressive and permanent in up to 25% of all cases [1], [2], [3].

Similar to the optic nerve, the olfactory nerve (ON) is covered by a meningeal sheath enclosing the subarachnoidal space. Elevated intracranial pressure (ICP) is a characteristic feature of IIH and could damage the olfactory nerves (ONs) directly by mechanical impact. There are also case reports about nasal liquor leakage in IIH patients [4], [5]. The authors argue that an increased ICP may break the nerve sheaths around the olfactory nerves that allow for liquor passage via the cribriform plate. In addition, there is growing evidence that an extensive lymphatic network system around the ONs could play a role in CSF absorption. The pathway of CSF absorption leads along the ONs and the absorbing acting system is located in the submucosal space associated with the nasal olfactory and respiratory epithelium [6]. The hypothesis that patients with IIH suffer from hyposmia has been suggested by Kapoor [7]. Giuseffi and colleagues reported that up to 25% of IIH patients complain about decreased smell [8]. This assumption is clinically relevant, since undetected and therefore untreated olfactory disorders are associated with reduced quality of life and problems with daily life situations [9], [10]. Furthermore, patients with hyposmia are at higher risk to develop depression [11]. However, to the best of our knowledge, clinical studies investigating the ON system in patients with IIH have not been reported in the literature.

Decreased olfactory function is mostly associated with reduced olfactory bulb volume (OBV) [12], [13], [14], [15]. Buschhüter et al. investigated a large cohort of normal volunteers and defined normative values for minimal-normal OBV as 58 mm^3^ in people <45 years and as 46 mm^3^ in people >45 years [15]. The importance of the determination of the depth of olfactory sulcus (OS) is less well known. Previous work has indicated a correlation between reduced olfactory function and smaller depth of OS in patients with olfactory dysfunction since birth or early childhood [16]. Wang and colleagues have demonstrated that the OS depth is reduced in patients with Parkinson disease [17].

The aim of our pilot study was to investigate OBV and OS depth to verify if the ON system is affected in IIH.

## Methods

Patients fulfilling the modified Dandy criteria for IIH [18] and an age over 18 years were screened using the hospital’s electronic medical records system. Patients with any secondary cause of intracranial hypertension were not eligible for inclusion in the study. Screening period was from November 2005 until May 2010. Approval for this study was obtained from the institutional ethics committee (Ethikausschuss 1, Charité Campus Mitte). Written informed consent was obtained from all participants before enrolment in the study. All clinical investigations have been conducted according to the principles expressed in the Declaration of Helsinki. Seventy-one patients were potentially eligible to be included in the study. Altogether 15 patients were excluded by the exclusion criteria shunt surgery (n=6), body weight over 180 kg (n=3), pregnancy (n=1), and magnetic resonance imaging (MRI) phobia (n=4). One patient fulfilled the criteria for major depression (diagnosed by the Becks Depression Inventory and Hamilton Rating Scale for Depression) and was excluded. Eighteen patients could not be reached by phone, eight patients refused because effort of participation in the study was too high for them, and seven subjects objected to participation without specifying any reasons. All participants underwent clinical examination to detect olfactory disorders with a different genesis (e.g. post-infectious, post-traumatic, current sinunasal or upper respiratory tract infections, tumors treated with radiation or chemotherapy, allergies, depression) at the Special Consulting Service for Olfactory Disorders at the ENT-Department of the Charité University in Berlin, Germany. Altogether, 23 patients could be included in the study.

**Figure 1:**
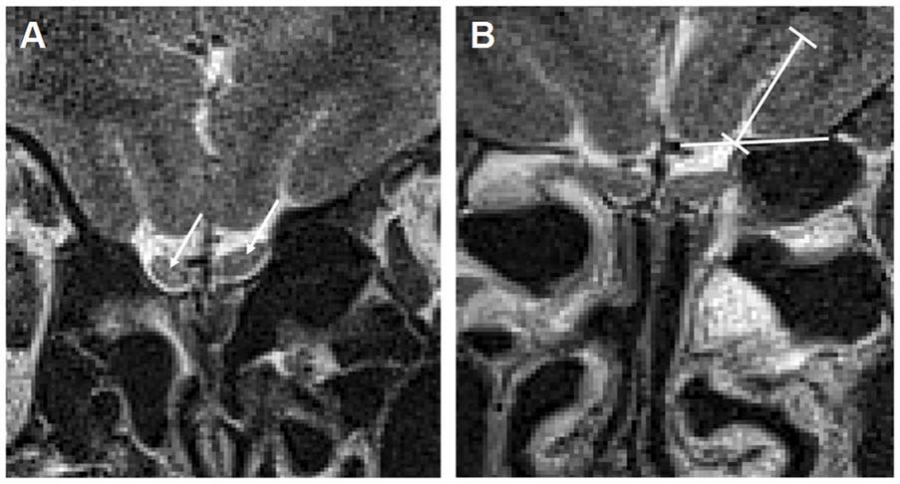
T2-weighted high-resolution coronal images of the olfactory bulb and sulcus olfactorius. Figure 1A and 1B show T2-weighted fast spin echo (FSE) sequences. In Figure 1A the white arrows indicate the normal dimensioned right and left bulb olfactorius. Figure 1B demonstrates the calculation of the olfactory sulcus (OS) depth. The distance of the deepest point of the OS was determined using a tangent line from the border of the gyrus rectus to the internal orbital gyrus.

Each patient with IIH was matched with a control patient for sex, age and body mass index (BMI). Controls were selected from a local obesity center and from the hospital staff. Subjects with known history of CNS disease or episodes of continuous or recurring headache syndromes were not eligible to be included in the control group.

MRI was performed with a 1.5 T scanner (Siemens, Magnetom). MRI was obtained to exclude intracranial pathology and sinus vein thrombosis as a secondary cause of IIH. A commercially available surface coil (Siemens) with a diameter of 7 cm was used in addition to the normal circularly polarized head coil. The surface coil was placed over one eye within the head coil and fixed with tape. T2-weighted high-resolution coronal images were acquired with the surface coil. A fast spin echo (FSE) sequence with a relaxation time of 6960 msec, an echo time of 99 msec, a field of view of 85×85 mm^2^, a matrix size of 256×256 mm^2^ (in plane resolution 332×332 µm^2^) and a slice thickness of 2 mm were performed. The measured time scale was 7 minutes and 20 seconds. The OBV as well as the OS depth were determined using a standardized method [19]. Computer software Amira 3.2 was used to calculate quantitative morphological parameters. OBV was investigated by circumnavigating the bulb contours of all coronal slices starting from the anterior to the posterior border of the olfactory bulb. The slice thickness was 2 mm, therefore the OBV could be calculated using the surface areas of each coronal section through the olfactory bulb (surface area of each coronal section in mm^2^ × count of sections × 2 mm). The OS depth was identified at the level of the last coronal slice through the rearmost part of the eyeball. The depth of the OS was then calculated by drawing a straight line tangent to the borders of the straight gyrus and internal orbital gyrus. From this line an intergyral line to the deepest point of the OS determined the OS depth (Figure 1). MRI study readers were blinded to the status and clinical characteristics of the participating subjects to prevent observer-dependent bias.

**Table 1:** Characteristics of participants and results of morphological features of olfactory nerve system.

	Patients n=23 (all)	Controls n=23 (all)	Significance *p*	Patients n=8 (IIH<1 year)	Controls n=8	Significance *p*
Gender, f/m	20/3	20/3	1	7/1	7/1	1
Age, years	37.0±13.7(20–63)	37.8±12.0(22–61)	0.85	33.5±9.9(20–48)	33.6±9.0(22–49)	0.98
BMI, kg/m2	33.5±7.7(24.1–54.6)	33.6±7.1(25.5–49.1)	0.96	33.1±10.0(25.9–54.6)	33.4±8.4(25.5–49.1)	0.95
Diagnosis of IIH*, month	34.1(0.4–121.0)	–	–	2.4 (0.4–12)	–	–
Headache, n(%)	16(69.6)	–	–	7(87.5)	–	–
Visual disturbance, n(%)	21(91.3)	–	–	6(75)	–	–
Medication, n(%)	16(69.6)	–	–	7(87.5)	–	–
Right OBV, mm^3^	63.2±21.6(37.0–124.1)	65.6±20.3(38.8–128.1)	0.69	55.7±15.6(40.1–82.9)	72.3±15.9(54.5–98.1)	0.05
Left OBV, mm^3^	65.5±19.4(38.4–103.0)	64.4±16.6(24.0–88.2)	0.84	60.9±13.2(38.4–80.3)	70.0±9.9(66.1–88.2)	0.01
**Total OBV, mm^3^**	**128.7±38.4(78.1–227.1)**	**130.0±32.6(72.5–202.8)**	**0.90**	**116.6±24.3(78.5–153.9)**	**149.3±22.2(124.4–186.3)**	**0.01**
Right OS, mm	8.6±1.3(6.3–11.8)	8.8±1,4(6.3–12.2)	0.69	8.8±1.9(6.3–11.8)	9.0±1.1(7.7–11.1)	0.80
Left OS, mm	8.4±1.4(4.9–11.1)	8.3±1.3(6.3–10.6)	0.83	8.6±2.1(4.9–11.1)	8.6±1.2(6.3–10.1)	0.99
Average OS, mm	8.5±1.2(5.8–11.5)	8.6±1.1(6.3–10.8)	0.91	8.7±1.8(5.8–11.5)	8.8±0.9(7.0–9.7)	0.89

Numerical data are presented as mean ± standard deviation (minimum-maximum)*, categorical data as numbers (n) and percent (%). The chi-square test was used for gender and independent T-test was used for numerical variables. Diagnosis of IIH=time between onset of first symptoms and enrolment in the study. Highest and last ICP=Highest and last intracranial pressure in the medical history of patients. CSF=Cerebrospinal fluid. Medication = Current intake of acetazolamide, topiramate or furosemide to treat IIH. OBV=Olfactory bulb volume. OS=Olfactory sulcus. *Numerical data of diagnosis of IIH are presented as median (range: minimum-maximum) because the data were not normally distributed.

Data were presented as mean ± standard deviation (range) or as median (range), if they were not normally distributed. The chi-square test and independent *t-*test were used to analyse differences between IIH patients and controls. To determine age-dependent normal OBV, data proposed by Buschhüter et al. (≥58 mm^3^ in people <45 years and ≥46 mm^3^ in people >45 years) were used [15]. Relationships among clinical features of IIH patients, the OBV, and the depth of OS were examined by using Pearson correlation coefficient r. A difference was considered significant at a *p-*value of <0.05.

**Figure 2:**
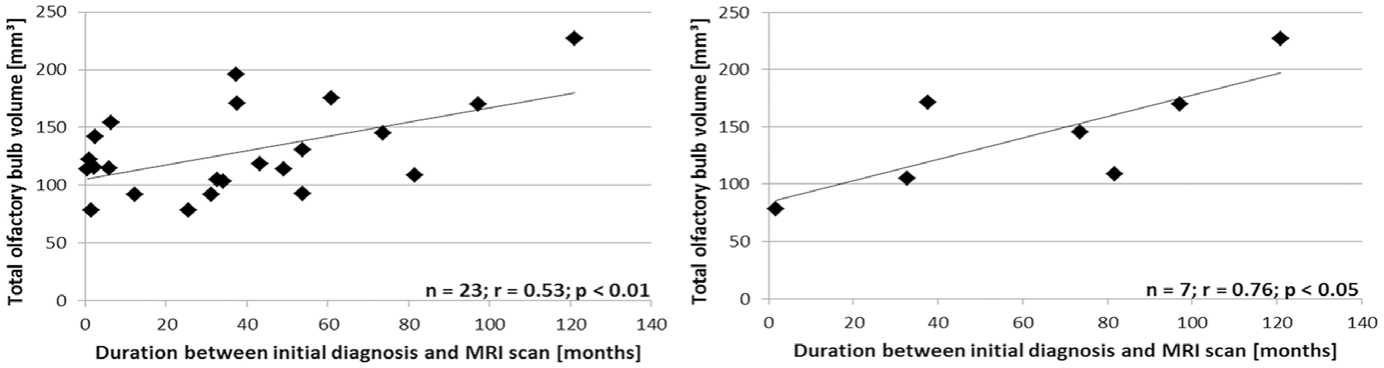
Correlation among clinical features of IIH patients. Left chart, correlation between total OBV and the time between initial diagnosis of IIH and MRI scan in all IIH patients (n=23). Right chart, Pearson correlation between total olfactory bulb volume (OBV) and the time between initial diagnosis of IIH and MRI scan in untreated symptomatic patients (n=7).

## Results

Twenty-three patients with IIH and 23 sex, age and BMI matched controls were included in the study. Twenty participants of each group were female. Patients had a mean age of 37 years, and the mean BMI was 33.5 kg/m^2^. There was no significant difference between patients and controls regarding gender, age, and BMI. Median time between onset of symptoms and begin of the study was 34 months. Medical history revealed a mean highest ICP of 37.6 cm CSF in recumbent position. Last measured ICP was still elevated with a mean of 28.8 cm CSF. The median time interval between the last measurements of ICP in IIH patients and the MRI study was 7.6 months. Headache was present in 16 (69.6%) and visual disturbance in 21 (91.3%) patients at the time of the scan. Sixteen (69.6%) patients were on an IIH treatment with acetazolamide, topiramate or furosemide (Table 1). Seven patients were not on medication at time of MRI. All of the untreated patients were still symptomatic (headache or visual disturbance present). While mean values of all morphological parameters were nearly similar in both groups, Pearson correlation showed that patients with a shorter medical history of IIH revealed a smaller OBV (r=0.53, *p*<0.01). In untreated symptomatic patients (n=7), the effect was greater (r=0.76, *p*<0.05) (Figure 2). The significance of the results was maintained after correction for age of patients. Another analysis revealed that in patients who suffered from IIH for less than one year (n=8), total OBV was significantly smaller than in matched controls (116.6±24.3 vs. 149.3±22.2 mm^3^, *p*=0.01)(Table 1). Seven olfactory bulbs (43.8%) revealed reduced OBV in the group of patients with IIH for less than one year, while no matched control subject had a reduced OBV (*p*<0.01). Compared with patients who suffered from IIH for more than one year (n=15) the difference of total OBV failed to reach significance (116.6±24.3 vs. 135.1±43.6 mm^3^, *p*=0.21) and there was no difference in number of reduced OBV (7/16 bulbs (43.8%) vs. 9/30 bulbs (30.0%), *p*=0.38). The median time interval between the last measurements of ICP in patients with IIH for less than one year and the MRI study was 1 month. Compared to patients who suffer for more than one year from IIH, last measured ICP was nearly similar compared to the group with IIH for less than one year (28.0±7.0 cm vs. 30.4±2.6 cm CSF, *p*=0.27). During the period that preceded study inclusion, the mean ICP decreased significantly in all IIH patients (37.7±6.8 vs. 28.9±5.9 cm CSF, *p*<0.001). In the group of untreated symptomatic patients, the ICP was also decreased during the period prior to inclusion in the study (36.1±5.6 vs. 29.5±7.3 cm CSF, *p*<0.001).

OS depth was similar in IIH patients and controls to an earlier published trial of healthy volunteers [14]. IIH patients with visual disturbances at time of MRI scan (n=21) revealed a lower OS depth compared to patients without visual disturbances (8.3±0.9 vs. 10.8±1.0, *p*<0.01). No other significant correlations were found.

## Discussion

The aim of our pilot study was to investigate whether patients with IIH exhibit morphological changes of the ON system. From the pathophysiological point of view, it is conceivable that elevated ICP could compromise the ON. Furthermore, an extensive lymphatic network system around the olfactory nerves seems to play a potential role in CSF absorption. Altered lymphatic dynamics within the nasal compartment may at least partially contribute to ON changes.

We investigated 23 IIH patients and 23 matched controls. The median time interval between diagnosis of IIH and MRI was 34 months and around 70% of IIH patients were treated. This might be responsible for finding no difference between the main groups of our pilot study. Short duration of IIH was associated with smaller amounts of total OBV even in untreated symptomatic patients. All IIH patients had a history of significantly reduced ICP during the period that preceded study inclusion. This might be caused by the course of disease in combination with the pharmacotherapy. Therapeutic weight loss and therapeutic lumbar punctures should be also taken under consideration, even in untreated IIH patients without pharmacotherapy at time of MRI. Due to the considerable regenerative ONS potential [20], [21], it is conceivable that its reconstitution might have already been induced by subtle ICP decreases. Changes of the ONs could therefore be expected in an earlier stage of IIH which is frequently associated with higher ICP levels.

Our study revealed a number of limitations. The time interval between last ICP measurements in all patients and MRI was with 7.6 months too long. In an optimal setting it should be shorter. However, from an ethical point of view it would be questionable to perform another lumbar puncture in order to improve the design.

The group patients with duration of IIH for less than one year revealed significantly reduced OBV compared to matched controls. In the smaller IIH group of eight patients the median time interval between last ICP measurements and MRI was with one month markedly shorter.

The distribution of sex, age and BMI (female to male ratio: 20∶3, mean age: 37 years, mean BMI: 33.5 kg/m^2^) in our study is comparable to other study cohorts. Boll et al. reported in a review that IIH is most common in women and obese individuals. The published female to male ratios range from 4∶1 to 15∶1 and the frequencies of obesity in IIH patients are 71%, 88%, 91%, and 94% [1]. Dhungana and colleagues indicated in a review that IIH most commonly affects overweight women between the ages of 15 and 45 years [2]. Out of 71 potentially eligible IIH patients 23 were included. 15 patients were excluded by the predefined exclusion criteria like conditions after shunt surgery or MRI phobia. A large proportion of the potentially eligible IIH patients could not be contacted by phone or participation expense were too high for them. Taking this into account, we cannot rule out the probability that patients with reduced social activities or even with depression might have refused participation in the study. Nevertheless, subjects suffering from depression for example tend to present more often with olfactory disorders as compared to the general population [22]. Among other things, OBV is dependent from neuroblast migration from the lateral ventricular extension of the forebrain. OBV seems to be dependent on input of smelling and it could be demonstrated that migration velocity depends on sensory input [20], [23].

Our pilot study suggests the possibility that morphological changes of the ON system could be present in IIH patients, especially with a short duration of disease. As it is well known that the OBV decreases with increasing age [15], it is unlikely that we have presented a natural course of aging. Nevertheless, a prospective longitudinal study design starting from the onset of the disease is required to confirm our results.

Patients with visual disturbance revealed a lower depth of OS. Whether the reduced OS depth in patients with visual disturbances is functionally relevant, remains unclear. Increased ICP or disturbed CSF dynamics could lead to a morphological alteration without functional correlate. Olfactory testing is necessary to answer the question, whether patients with IIH suffer from hyposmia, especially in the early stages of disease. The UPSIT (University of Pennsylvania Smell Identification Test) or the TDI (threshold, discrimination and identification) test using “sniffin’ sticks" [24], [25] could be useful tools to clarify this question. Although, decreased olfactory function is associated with reduced olfactory bulb volume (OBV) [12], [13], [14], [15], testing of olfactory capacity can not be replaced by the measurements of OBV or OS depth. Therefore, further trials are required to investigate the olfactory system in IIH patients.
